# Early Microglia Activation Precedes Photoreceptor Degeneration in a Mouse Model of CNGB1-Linked Retinitis Pigmentosa

**DOI:** 10.3389/fimmu.2017.01930

**Published:** 2018-01-05

**Authors:** Thomas Blank, Tobias Goldmann, Mirja Koch, Lukas Amann, Christian Schön, Michael Bonin, Shengru Pang, Marco Prinz, Michael Burnet, Johanna E. Wagner, Martin Biel, Stylianos Michalakis

**Affiliations:** ^1^Institute of Neuropathology, Faculty of Medicine, University of Freiburg, Freiburg, Germany; ^2^In Vivo Pharmacology, Synovo GmbH, Tübingen, Germany; ^3^Center for Integrated Protein Science Munich CiPSM and Department of Pharmacy, Center for Drug Research, Ludwig-Maximilians-Universität München, Munich, Germany; ^4^Faculty of Biology, University of Freiburg, Freiburg, Germany; ^5^Institute for Medical Genetics and Applied Genomics Transcriptomics, University of Tübingen, Tübingen, Germany; ^6^IMGM Laboratories GmbH, Planegg, Germany; ^7^BIOSS Centre for Biological Signalling Studies, University of Freiburg, Freiburg, Germany

**Keywords:** retinitis pigmentosa, retinal degeneration, cyclic nucleotide-gated channel, microglia, innate immune response

## Abstract

Retinitis pigmentosa (RP) denotes a family of inherited blinding eye diseases characterized by progressive degeneration of rod and cone photoreceptors in the retina. In most cases, a rod-specific genetic defect results in early functional loss and degeneration of rods, which is followed by degeneration of cones and loss of daylight vision at later stages. Microglial cells, the immune cells of the central nervous system, are activated in retinas of RP patients and in several RP mouse models. However, it is still a matter of debate whether activated microglial cells may be responsible for the amplification of the typical degenerative processes. Here, we used *Cngb1*^−/−^ mice, which represent a slow degenerative mouse model of RP, to investigate the extent of microglia activation in retinal degeneration. With a combination of FACS analysis, immunohistochemistry and gene expression analysis we established that microglia in the *Cngb1*^−/−^ retina were already activated in an early, predegenerative stage of the disease. The evidence available so far suggests that early retinal microglia activation represents a first step in RP, which might initiate or accelerate photoreceptor degeneration.

## Introduction

It is generally accepted that immune responses follow injury and damage to tissues and organs. Microglia are the resident immune cells within the brain and retina, commonly known as the macrophages of the central nervous system (CNS). In response to injury or inflammatory stimuli, the resting microglia can be rapidly activated to participate in pathological responses, including migration to the affected site, release of various inflammatory molecules, and clearing of cellular debris (1–3). Although microglia are essential for maintaining a healthy CNS, paradoxically they may undergo phenotypic changes to influence several neurodegenerative diseases and psychiatric disorders including Alzheimer’s disease (AD), Parkinson’s disease, and Rett syndrome (4). Moreover, activation of microglia has also been detected in several retinal degenerative mouse models (5, 6) and in patients suffering from retinitis pigmentosa (RP) (7). RP describes a heterogeneous group of hereditary retinal degenerations with a world-wide prevalence of 1:4,000 (8). To date, more than 50 different genetic mutations have been detected, which cause non-syndromic RP (9). RP is characterized by an initial progressive degeneration of rods and followed by the loss of cones leading to severe visual impairment (8, 10). It should be noted that the disease severity, rate of disease progression, age of onset and clinical findings may differ significantly among patients based on the fact that RP represents a heterogeneous group of inherited retinal disorders (11). Typically, the earliest clinical symptom of RP is an initial night blindness caused by the dysfunctional rod system. Subsequent degeneration of cones leads to a gradual loss of the visual field, which initially impairs the periphery and spreads to the macula. The consequences include so-called “tunnel vision” and eventually complete blindness (10).

Here, we used the *Cngb1* knockout (*Cngb1*^−/−^) mouse to study the activation of immune cells in a model of RP with slowly progressing photoreceptor degeneration. *Cngb1* encodes the B subunit of the cyclic nucleotide-gated channel in rod photoreceptors. *Cngb1*^−/−^ mice show initial signs of rod degeneration including gliosis already between 14 and 21 days of age (12) while the peak of neuronal cell death occurs around 4 weeks of age (13). Even though the degenerative process begins already at this early age, the degeneration advances very slowly and shows a slower progression of the disease when compared with other RP mouse models like the rd1 mice (14). For RP a general feature is that cone photoreceptors deteriorate secondary to rods with a considerable slower progression rate (12). In the present study, we found that microglia in the *Cngb1*^−/−^ retina showed already increased cell numbers and pronounced activation in 4-week-old mice. At this time point only a minor photoreceptor cell loss was detected. Our data suggest that *Cngb1*^−/−^ microglia are potentially an early driving force, which substantially contributes to the retinal degeneration and long-term visual impairments found in RP.

## Materials and Methods

### Animals

*Cngb1*^−/−^ were generated by us (12). All mice used in the study were bred on a mixed genetic background of the 129Sv and C57BL/6N strain. Animals were housed under standard white light (200 lux, 12 h dark–light periods) with free access to food and water. Both male and female mice were used in equal shares. Age-matched wild-type mice were used as controls. Day of birth was considered as postnatal day 1 (P1). All procedures concerning animals were performed with permission of the local authority (Regierung von Oberbayern and RP Freiburg).

### Optical Coherence Tomography (OCT) Analysis

For OCT examinations, mice received intraperitoneal injections of ketamin (0.1 mg/g) and xylazin (0.02 mg/g). Before the scanning procedure, Tropicamid eye drops were instilled into the eye for pupil dilation (Mydriadicum Stulln, Pharma Stulln GmbH, Stulln, Germany). Subsequently, hydroxylpropyl methylcellulose (Methocel 2%; OmniVision, Puchheim, Germany) was applied to keep the eyes moist. The examination was performed with an adapted Spectralis HRA + OCT system by Heidelberg Engineering (Dossenheim, Germany) in combination with optic lenses described previously (15). OCT scans were conducted using a 12 circular scan mode centered at the optic nerve head. This procedure allowed for measurements of the photoreceptor layer thickness at a comparable distance from the optic nerve head. In detail, outer nuclear layer (ONL) thickness was measured between the clearly visible outer limiting membrane and the outer plexiform layer (OPL). For statistical analysis, the mean ONL thickness was calculated from single values measured in the dorsal, temporal, nasal, and ventral region around the optic nerve.

### Microarray Analysis

For microarray experiments, retinal tissue was obtained from mice of two different age groups (P12 and P28). For differential gene expression analysis of *Cngb1*^−/−^ and wt animals, an Affymetrix platform was used according to the manufacturer’s instructions as described before (16). In short, retinas were dissected, shock-frozen in liquid nitrogen and stored at −80°C until further use. RNA was extracted using RNeasy Minikit (Qiagen, Hilden, Germany) according to the manufacturer’s instructions. RNA concentration and purity were determined using NanoDrop2000 (Thermo Scientific^®^). Fragmented and labeled cRNA of three wild-type and three *Cngb1*^−/−^ retinas was hybridized on Affymetrix Mouse Genome 430 2.0 Arrays, respectively. A probe-level summary was determined with the help of Affymetrix GeneChip Operating Software using the MAS5 algorithm. Raw data were normalized using the Array Assist Software 4.0 (Stratagene, La Jolla, CA, USA) in combination with the GC-robust multichip average algorithm. Significance was determined by a *t*-test without multiple testing correction (Array Assist software), selecting all transcripts with a minimum change in expression level of 1.5-fold together with a *p*–value <0.05.

### Quantitative PCR

cDNA synthesis was performed with the RevertAid First Strand cDNA Synthesis Kit (Thermo Scientific) according to the manufacturer’s manual. PCR was performed on a StepOnePlus Real-Time PCR System (Applied Biosystems) using SYBR Select Master Mix (Applied Biosystems). For quantitative PCR, two technical replicates per gene were generated and normalized to the housekeeping gene aminolevulinic acid synthase. The following primer sets were used:

**Table:** 

Gene	Forward primer (5′→3′)	Reverse primer (5′→3′)
*Irf8*	GCTGATCAAGGAACCTTGTG	CAGGCCTGCACTGGGCTG
*Aif1/Iba-1*	ATCAACAAGCAATTCCTCGATGA	CAGCATTCGCTTCAAGGACATA
*C1qc*	CCCAGTTGCCAGCCTCAAT	GGAGTCCATCATGCCCGTC
*Cx3cr1*	GAGTATGACGATTCTGCTGAGG	CAGACCGAACGTGAAGACGAG

### Flow Cytometry

*Cngb1*^−/−^ and wt mice were euthanized at P28 and perfused with phosphate-buffered saline. Retinas were removed and mechanically dissociated into single cell suspensions by pipetting. Dissociated cells were stained with live/dead dye (1:1,000, eBioscience) in PBS for 30 min at 4°C. In order to prevent unspecific binding to Fc receptors, their binding domains were blocked by unstained CD16/32 (1:250, 2.4G2, Becton Dickinson) in FACS-Buffer (2% FCS, 5 mM EDTA in PBS) for 20 min at 4°C. Cells were stained with CD11b (BV421, 1:300, M1/70, eBioscience), CD45 (APC-eF780, 1:200, 30-F11, eBioscience), F4/80 (PE; 1:200, BM8, eBioscience), CD44 (PE, 1:200 IM7, Becton Dickinson), and MHC class II (PE, 1:200, M5/114.15.2, eBioscience) in FACS-Buffer at 4°C for 20 min and analyzed using a FACSCanto II (Becton Dickinson). Viable cells were gated by forward and side scatter pattern. Data were acquired with FACSdiva software (Becton Dickinson). Postacquisition analysis was performed using FlowJo software (Tree Star, Inc.).

### Retina Preparation and Immunohistochemistry

Retinas were dissected at P28 and further processed as described for immunohistology and whole mount preparation (17–19). Primary antibodies were added overnight at a dilution of 1:500 for Iba-1 (019-19741, WACO, Japan), 1:250 for Lamp 2 (ab13524, Abcam, Cambridge, UK), 1:200 for cleaved caspase-3 (9661, Cell Signaling Technology, Danvers, MA, USA) and 1:100 for Mhc class II (ab23990, Abcam), at 4°C. Secondary antibodies were added at the following dilution: Alexa Flour 488 1:500, Alexa Flour 555 1:500 and Alexa Fluor 568 1:500 for 2 h at room temperature. Nuclei were counterstained with DAPI. The examined area was determined microscopically by a TCS SP8 confocal scan microscope (Leica) or a conventional fluorescence microscope (Olympus BX-61).

### Visual Cliff

The visual cliff behavior was analyzed in an open-top Plexiglas chamber. Half of the box protruded from the counter to provide a 3-foot depth. The box on the counter displayed a base with a checkerboard pattern and the box off the counter showed the base with the same checkerboard pattern, except for the 3 feet of depth. The mouse was placed on the dividing line between both halves of the chamber at the edge of the counter and was allowed to choose between the two sides. If the mouse stepped to the shallow side, time was scored as time spent on the “safe side.” Each mouse performed this task twice for 10 min with a time window of 1 h between trials. The visual cliff behavior was averaged to generate mean percentage of time in which the mouse chose to stay at the shallow side (*n* = 3–5 mice per group).

### Statistical Analysis

All graphical data represent mean ± SEM. Sample sizes are provided in the figure legends. In order to test for significant differences, an unpaired *t*-test was applied. Differences were considered as significant when *p*-value <0.05.

## Results

**Figure 1:**
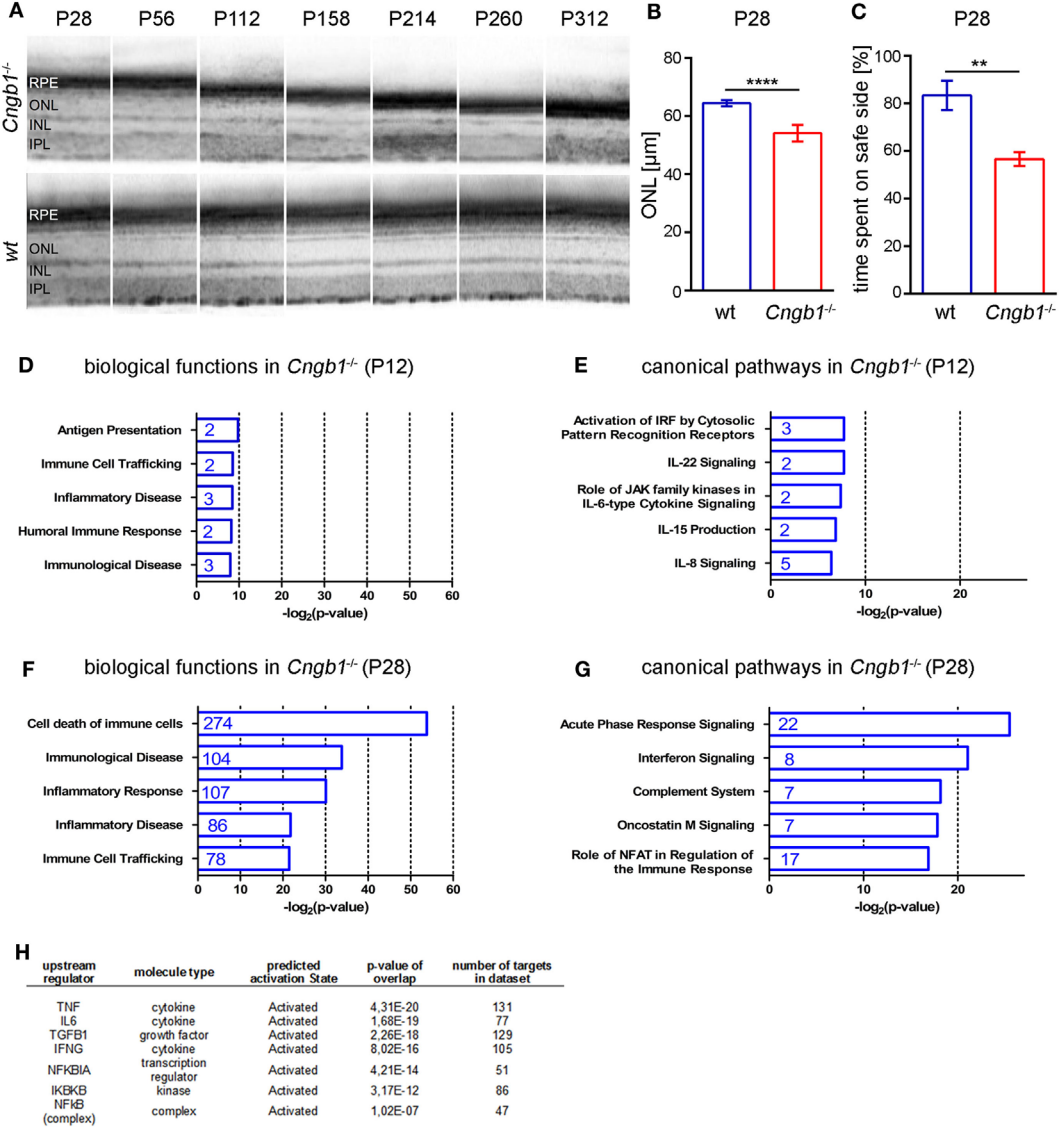
Activation of immunological pathways in *Cngb1*^−/−^ retinas at 12 and 28 days after birth. **(A)** Representative optical coherence tomography (OCT) images from *Cngb1*^−/−^ (upper panel) and wild-type retinas (lower panel), which display the slow progression (P28-P312) of outer nuclear layer (ONL) thinning over time. **(B)** Quantification of ONL thickness from OCT data at P28 (****p < 0.0001, *n* = 6 for each genotype). **(C)** Performance in the visual cliff test for the study of visual depth perception (**p < 0.01, *n* = 3 for each genotype). **(D–G)** Biological functions and canonical pathways were significantly altered in *Cngb1*^−/−^ mice compared to age-matched controls. A high number of genes were related to the immune system or to immune responses. **(H)** The indicated upstream regulators for proinflammatory cytokines and the NF-κB pathway were predicted to have a significantly higher activation state in *Cngb1*^−/−^ retinas when compared to age-matched wild-type controls (*p* < 0.05).

At 28 days after birth (P28), minor (~15%) but significant retina degeneration was observed in *Cngb1*^−/−^ mice (Figures 1A,B) (12) and more than 1,000 genes were dysregulated (>1.5-fold dysregulated, *p* < 0.05, STab.1) as seen from Affymetrix gene chip arrays. Already at this early time point *Cngb1*^−/−^ mice displayed substantial visual impairment (Figure 1C). We were analyzing gene expression data with the help of Ingenuity Pathway Analysis software, to identify potential shifts in biological functions or in canonical pathways at early and predegenerative stages. Interestingly, activation of the immune system was already apparent 12 days after birth (P12) as indicated by upregulated genes that were involved in processes like antigen presentation, immune cell trafficking, immunological diseases, humoral immune response, and inflammatory disease (Figures 1D,E). At P28 many of the dysregulated genes were attributed to cell death, survival, or neurological diseases and also to pathways and signaling cascades that are assigned to immunological processes (Figures 1F,G; Table S1 in Supplementary Material). Particularly genes linked to inflammatory responses, inflammatory diseases and immune cell trafficking were significantly altered in *Cngb1*^−/−^ retinas at P28 when compared to wt retinas. A detailed analysis of upstream regulators (URs), which are not directly altered in their expression level but are responsible for expression changes of their target genes, revealed the activation of diverse proinflammatory mediators like TNF, IL6, and NF-κB in *Cngb1*^−/−^ retinas at P28 (Figure 1H).

**Figure 2:**
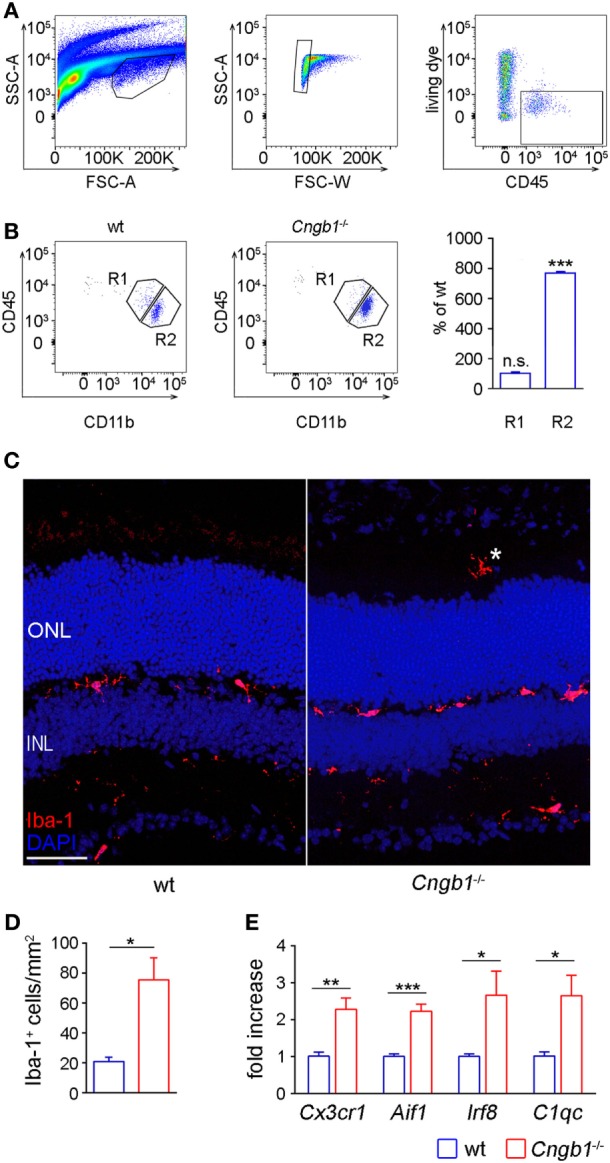
Strong increase of retinal microglia cells in *Cngb1*^−/−^ retinas 28 days after birth. **(A)** Gating strategy for identifying microglia cells in retina lysates by size (left panel), single cells (middle panel), and living CD45^+^ cells (right panel) expression. **(B)** Representative gating and quantification of CD11b^+^CD45^low^ retinal microglia (R2) and CD11b^+^CD45^hi^ cells (R1) from wild-type or *Cngb1*^−/−^ retinas. (Statistical significance was determined vs. percentage of wild-type cell counts, ns = non significant; ***p < 0.001, *n* = 3 for each genotype.) **(C)** Immunofluorescence of Iba-1 (red) in the retina of *Cngb1*^−/−^ and age-matched wt demonstrating migration of microglia into the photoreceptor layer of *Cngb1*^−/−^ mice (asterisk). Nuclei were counterstained with DAPI. ONL, outer nuclear layer, INL, inner nuclear layer, scale bar 50 µm. **(D)** Quantification of Iba-1-positive cells in *Cngb1*^−/−^ mice compared to wt animals (*p < 0.05, *n* = 4 for each genotype). **(E)** Gene expression of microglia-specific genes in *Cngb1*^−/−^ and wt retinas. Significant increase in the expression of *Cx3cr1, Aif1, Irf8*, and *C1qc* in retinas of *Cngb1*-deficient mice (*p < 0.05, **p < 0.01, ***p < 0.001, *n* = 4 for each genotype).

**Figure 3:**
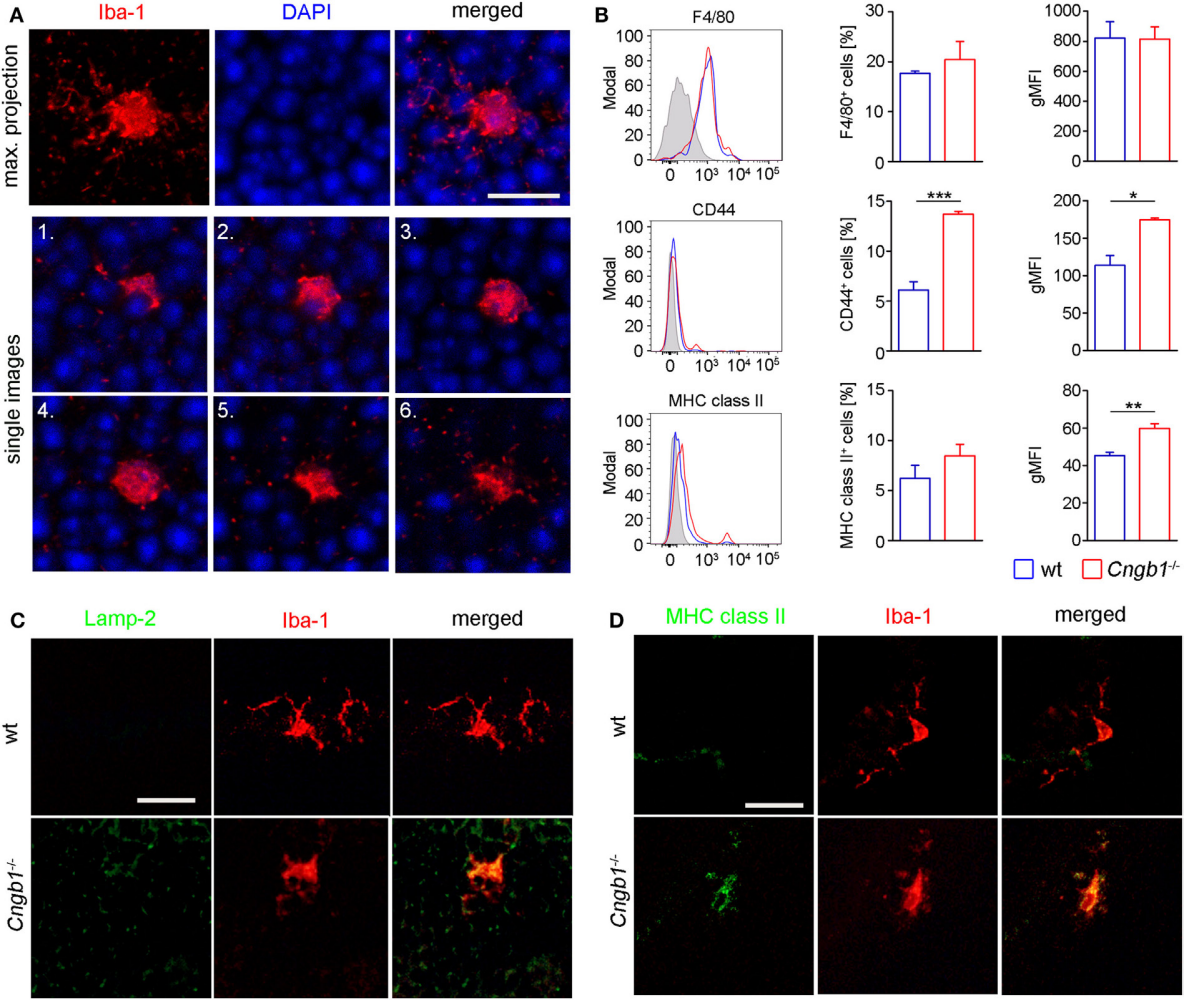
Amoeboid microglia morphology in *Cngb1*^−/−^ is accompanied by the expression of activation markers. **(A)** Whole mount images of Iba-1-stained microglia in the outer nuclear layer (ONL) of *Cngb1*^−/−^ mice induced morphological changes from a resting to an amoeboid phenotype at P28. Nuclei were counterstained with DAPI. Upper panel: maximum projection. Lower two panels: single images with an increment of 1.3 µm. Scale bar. 10 µm. **(B)** Flow cytometric analysis of retinal microglia for the expression of F4/80, CD44, and MHC class II (left panel). Quantification of the numbers (middle panel) and geometric mean fluorescent intensities (gMFI, right panel) of F4/80, CD44, and MHC class II are depicted. Results were obtained from two independent experiments with at least three replicates (*p < 0.05, **p < 0.01, ***p < 0.001, *n* = 3 for each genotype, blue line = wt, red line = *Cngb1*^−/−^, grey = isotype control). **(C)** Misplaced microglia in the ONL in *Cngb1*^−/−^ coexpressed activation marker Lamp-2. Scale bar 25 µm. **(D)** Confirmation of MHC II expression by costaining of Iba-1 (red) and MHC class II (green) in wt and *Cngb1*^−/−^ retinas. Scale bar 25 µm.

The generated microarray data clearly suggested the presence of an activated immune system in *Cngb1*^−/−^ retinas. That is why we focused on microglial cells, which represent the immune competent cells of the CNS and retina (5). Microglia tend to proliferate upon tissue destruction during neurodegeneration in order to clear the cellular debris and to restore tissue homeostasis (4). Thus, we first determined microglia cell numbers using flow cytometry (FACS), histological and qPCR approaches. For FACS analysis, we gated microglia as live CD45^lo^CD11b^+^ cells (Figures 2A,B). Quantification of single cell suspensions prepared from *Cngb1*^−/−^ and wt retinas 4 weeks after birth revealed significantly increased microglia cell numbers in *Cngb1*-deficient mice compared to age-matched wt (Figure 2B). CD45^hi^CD11b^+^ cell numbers were not increased (Figure 2B). Immunofluorescence of Iba-1, a specific marker for microglia and macrophages, confirmed a strong elevation of this immune cell population in retinas of *Cngb1*^−/−^ mice (Figures 2C,D). In addition to increased Iba-1^+^ cell numbers in degenerating retinas of *Cngb1*^−/−^ mice, microglial cells also changed their localization. In wt retinas, microglia cells were mainly found in the inner plexiform layer (IPL) or OPL, while Iba-1-positive cells of *Cngb1*^−/−^ retinas were additionally found in the ONL and in the photoreceptor layer close to the retinal epithelium (Figure 2C, asterisk; Figures 3A,C). Further analysis of wt and *Cngb1*^−/−^ retina microarray data indicated that several microglia-specific genes like *Cx3cr1, Aif1, Irf8, C1qc* (20–22) were upregulated in the *Cngb1*^−/−^ group (Table S1 in Supplementary Material). Subsequent RT-qPCR analysis of these microglia cell-specific genes confirmed their increased expression levels (Figure 2E). In summary, microglial cell numbers were strongly increased in *Cngb1*^−/−^ mice at 4 weeks of age, which corresponds to an early, degenerative stage of the disease.

**Figure 4:**
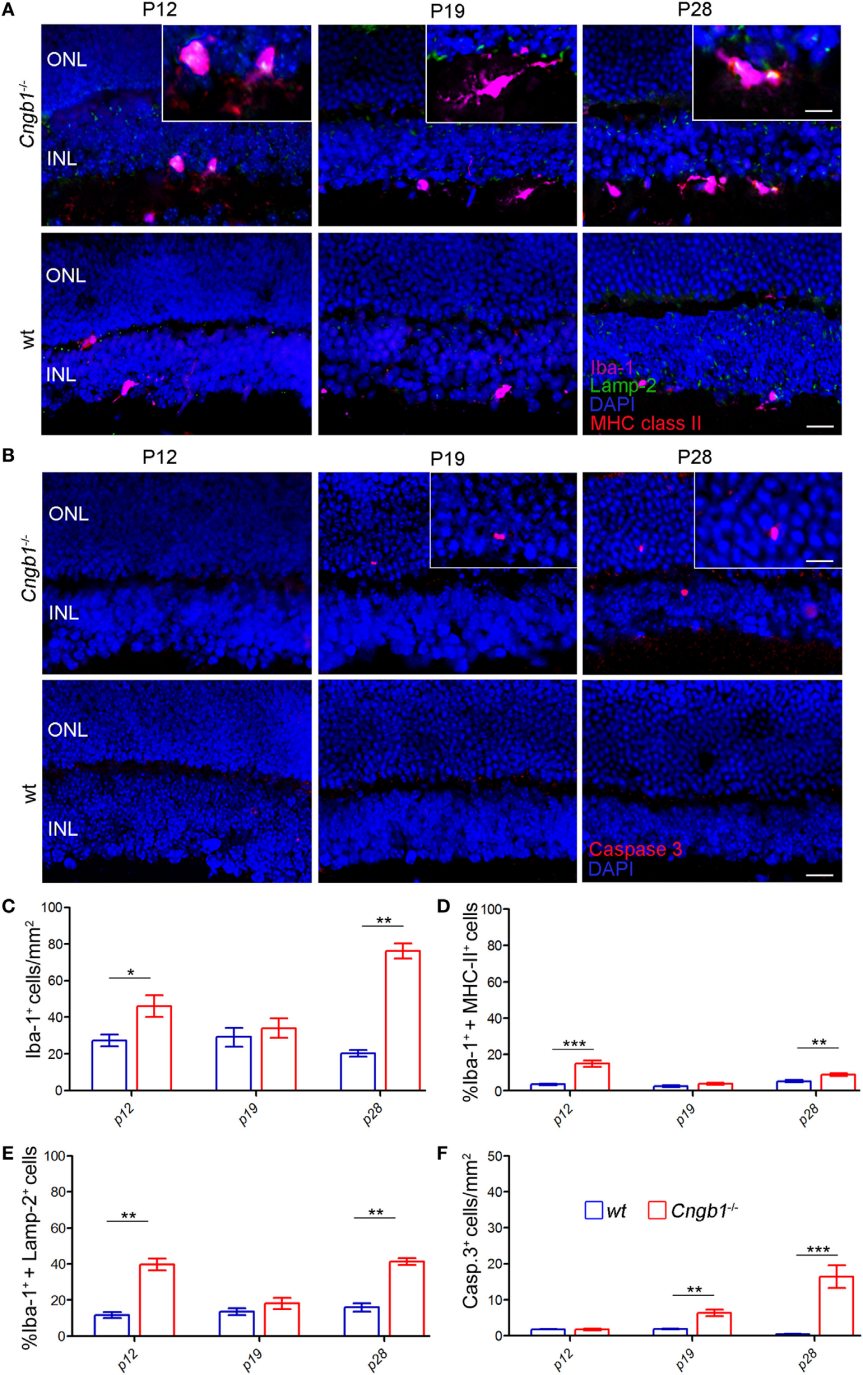
Time course of early photoreceptor apoptosis and microglia activation. **(A)** Costaining of Iba-1-positive microglia (pink) in the ONL and INL of *Cngb1*^−/−^ and wt mice at P12, P19 and P28 with the activation marker Lamp-2 (green) and MHC class II (red). **(B)** Immunofluorescence of cleaved caspase 3 (red) in *Cngb1*^−/−^ and wt mice. Nuclei were counterstained with DAPI (blue). **(C)** Quantification of Iba-1-positive cells and the percentage of Iba-1^+^ MHC class II− **(D)** or Iba-1^+^ Lamp-2-positive cells **(E)** in *Cngb1*^−/−^ mice compared to wt animals. **(F)** Cleaved caspase 3-positive cells in *Cngb1*^−/−^ and wt mice at indicated time points. ONL, outer nuclear layer, INL, inner nuclear layer, scale bar 20 µm; insert 10 µm (*p < 0.05, **p < 0.01, ***p < 0.001, *n* = 5 for each genotype).

In response to disrupted tissue homeostasis, microglial cells get activated and change their morphology together with the expression of surface markers (4). In *Cngb1*^−/−^ retinas, microglia showed a transition from a resting to an activated state (Figure 3A). The cells underwent morphological changes to take on an amoeboid shape with fewer branches compared to the resting state phenotype in wt retinas (23, 24). As specialized phagocytes, one of the functions microglia have is to remove debris of dying or dead cells (25). In mice, CD44 is a competent receptor for phagocytosis in macrophages (26) and an increase of CD44 expression was detected in the initial microarray data analysis (STab.1). Subsequent FACS expression analysis of CD45^lo^CD11b^+^ cells could link CD44 to microglia, as the number of CD45^lo^CD11b^+^CD44^+^ cells as well as the expression levels of CD44 in microglia in *Cngb1*^−/−^ retinas were elevated (Figure 3B). Active phagocytosis of microglial cells can also be monitored *in situ* by immunohistological staining of lysosome-associated membrane protein (lamp)-2 (19). In P28 *Cngb1*^−/−^ retinas, we detected Lamp-2-positive microglia particularly in the ONL and photoreceptor layer (Figure 3C). Increased MHC class II expression, which indicates microglial activation, was further observed by FACS and immunohistochemistry in *Cngb1*^−/−^ retinas when compared to wt retinas (Figure 3D), while the expression levels of the macrophage marker F4/80 remained unchanged in both genotypes (Figure 3B). Although the neurodegenerative process became evident at P28, microglia were activated already at P12 (Figures 4A,C–E). At this time point no apoptotic cells as indicated by the absence of positive signals for cleaved caspase 3 were present (Figures 4B,F).

**Figure 5:**
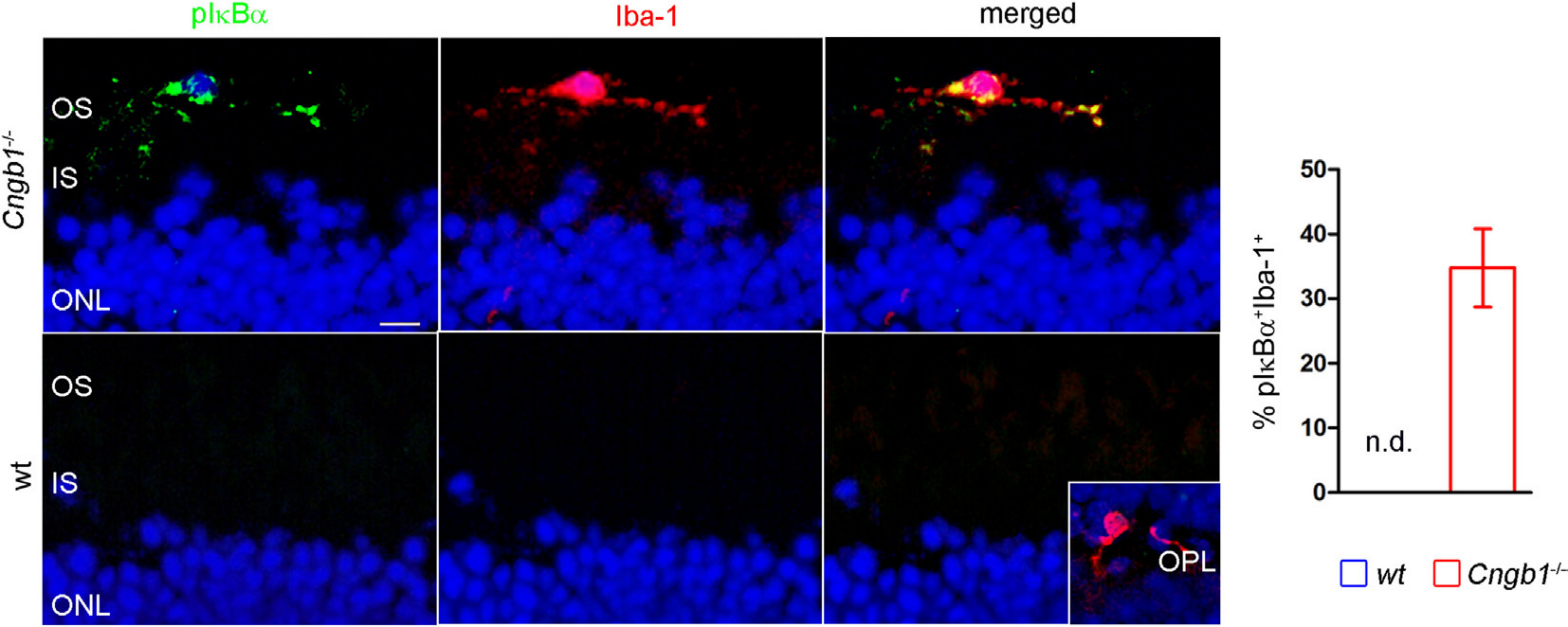
Activation of proinflammatory signaling in *Cngb1*^−/−^ mice. Immunofluorescence of Iba-1 (red) and pIκBα (green) in *Cngb1*^−/−^ and wt mice at P28. Quantification of double-positive cells in wt and KO revealed exclusive presence of active NF-κB-signaling in *Cngb1*^−/−^ mice (*n* = 3 for each genotype). Insert: Iba-1-positive resting microglia in the OPL. Nuclei were counterstained with DAPI. ONL, outer nuclear layer, IS, inner segment, OS, outer segment, OPL outer plexiform layer, scale bar 20 µm, nd = not detectable.

The Ingenuity UR analysis indicated increased activity of diverse proinflammatory signaling cascades (Figure 1H). One of these cascades was the NF-κB pathway, which can be induced by a variety of signals to finally induce a specific pattern of transcription. In this classical pathway, activated IKK-β, which is part of an IKK-α–IKK-β–IKK-γ complex, phosphorylates the inhibitory subunits IkB-α, IkB-β, or IkB-ϵ, leading to their proteasomal degradation. As a result, NF-κB homodimers and heterodimers, mainly composed of RelA, RelC, and p50, accumulate in the nucleus (27). Here, we confirmed the presence of an activated NF-κB-signaling pathway in microglia by the immunofluorescent detection of phosphorylated IκB colocalized with Iba-1-positive microglia, which were misplaced in the outer segment of *Cngb1*^−/−^ retinas (Figure 5).

## Discussion

Our present results link retinal degeneration to immune system activation, and here, more precisely, to the activation of microglia. In *Cngb1*^−/−^ mice, neuronal cell death does not start before postnatal day 15 (P15) (12). Already before this early stage of degeneration, retinal gene expression analysis at P12 indicated an immune response in biological and canonical pathways. These data clearly indicated that activation of the immune system starts prior to the actual retinal degenerative process in *Cngb1*^−/−^ mice. Between P21 and P28 retinal degeneration reaches its maximum (12). We determined activation of the immune system by gene expression pathway analyses and immunohistochemical detection. At this early disease stage, microglia had already migrated entirely through the various layers of the *Cngb1*^−/−^ retina toward the photoreceptors. Microglial cells are the local immune cells of the CNS and normally reside at the IPL/OPL of the retina (5, 28). Upon activation, microglia migrate toward the injury site, change their morphology from ramified cells to amoeboid phagocytes and start expressing several surface markers including F4/80, MHCII, and complement receptor 3 (CD11b/18, Ox42) (1, 5, 29). Our findings suggest that microglial activation occurs before the onset of neurodegeneration. This early microglia activation might be responsible for the observed high CD44 representation in *Cngb1*^−/−^ retinas. CD44 is implicated in the pathogenesis of inflammation and contributes to the recruitment of inflammatory cells as well as to increased phagocytosis (26, 30, 31). Increased relative expression of the cell surface adhesion receptor CD44 seems to be a very general feature of retinal degeneration considering that it was also present in rd10 mice (32). Previous work using the rd10 mouse model of RP had already suggested a contribution of microglia in retinal degeneration (33). In this mouse model activated microglia infiltrate into the photoreceptor layer and contribute actively to photoreceptor demise *via* the phagocytotic clearance of viable photoreceptors and the secretion of proinflammatory cytokines that potentiate photoreceptor apoptosis (33, 34). It still remained unclear whether microglial activation was responsible for further photoreceptor cell death. Even though, genetic depletion of microglia slowed down the degenerative process in rd10 mice (33). In follow-up experiments, it would be interesting to investigate whether microglial cells are actually the main detrimental force in *Cngb1*-deficient retinas. This could either be achieved by allowing CX3CR1^+^ retinal microglia to express diphtheria toxin and be specifically ablated upon tamoxifen administration (33, 35) or by pharmacological ablation using the CSF1R inhibitor (36).

Our results indicate that microglia activation is an important step in the degenerative process of rods in RP. The intriguing question however is whether microglia get activated during a predegenerative state or whether signals from a small number of degenerating cells is sufficient to initiate the activation of microglia before the actual “degeneration peak.” In the rd10 retina activated microglia infiltrate the ONL at P16. Since photoreceptor apoptosis started only at P19 microglia activation preceded the initiation of photoreceptor apoptosis (37). Comparable findings of microglia proliferation and activation at early time points were also described in rd1 and rd10 mice, which represent further mouse models of RP (37–39). Both RD models are induced by a mutation in the rod photoreceptor-specific Pde6b gene (40, 41). In fact, retinal architecture in rd10 mice displayed alterations from as early as P5, which is at least 13 days before photoreceptor loss (39). These alterations included increased proliferation of microglia within the retina, which ultimately led to increased numbers of activated microglia. At the same time there was a significant decrease of glutamine synthetase in Müller glia followed by an increase in glial fibrillary acidic protein immunofluorescence, which is expressed in Müller glia and astrocytes (39). A similar activation of astrocytes might be present in *Cngb1*-deficient retinas when one considers the abundant LAMP-2 labeling outside microglial cells. To what extent this reactive gliosis contributes to photoreceptor degeneration in both mouse models is not clear. The observed microglia activation can play a critical role in neuroinflammation and impose subsequent damage with progressive photoreceptor loss (42). In contrast, microglia might also be beneficial during retinal degeneration. This assumption is based on studies showing that microglia-derived trophic support protects photoreceptors *in vivo* under stressful conditions (43). Our data suggest that resident microglia and not monocyte-derived macrophages are mainly involved in the neurodegenerative process. Both cell populations are phenotypically distinguishable with a unique microglial CD45^lo^ CD11c^lo^ F4/80^lo^ I-A/I-E^lo^ profile and a monocyte-derived macrophage CD45^hi^CD11b^hi^ signature (44). However, it has also been shown that activated retinal microglia upregulate CD45 (45) and that differentiation of monocytes into macrophages may be associated with downregulation of CD45 sometimes to levels that make the two cell populations indistinguishable (46). The small CD45^hi^ CD11b^−^ cell population found in *Cngb1-*wt and *Cngb1-*ko retinas presumably represents circulating retina-specific T cells (47), which have been reported to protect against spontaneous organ-specific autoimmunity (48). At the molecular level, inflammation is often regulated by numerous molecules and factors, including the transcription factor NF-κB (49). The activation of NF-κB in microglia, as seen in our present RP mouse model, is often associated with the release of reactive oxygen species and proinflammatory cytokines (such as IL-1β, interferon-γ, and TNF-α) that can cause secondary neurotoxicity and neuronal cell death including the degeneration of photoreceptors (50). Dying photoreceptor cells, in turn, induce NF-κB in microglial cells and thereby further their activation (51). Detrimental NF-κB-signaling in microglia has a key role in several degenerative processes of the CNS as documented for aging including AD (52), amyotrophic lateral sclerosis (53), and multiple sclerosis (54). When mice and rats express mutant rhodopsin, they experience photoreceptor cell death and, much as humans, develop the clinical signs of autosomal dominant retinitis pigmentosa (ADRP). During the progression of ADRP, microglia get activated and display heightened NF-κB-signaling (55). Increased expression of NF-κB protein and NF-κB DNA-binding activity in microglia of the retina has also been reported during photoreceptor degeneration of *rd* mice. In this model, the neurotoxic role of microglial NF-κB activation in photoreceptor apoptosis was mediated by increased TNF-α production in microglial cells (56). Several studies have also indicated that NF-κB activation leads to enhanced IL-1β secretion by microglia, which makes them contribute to rod degeneration in RP by potentiating apoptosis (33).

In terms of therapy, targeting microglia may reduce the production of several proinflammatory mediators and may therefore result in broader therapeutic effects than inhibition of single cytokines. However, chemical or genetic depletion of microglia would provide an approach with only short-term beneficial effects since microglia has been shown to repopulate once the treatment ends (35, 36). Particular attention should be paid to unwanted depletion or damage to other cells like optic nerve oligodendrocyte precursor cells. As an example, secondary to microglia depletion by the CSF-1R inhibitor BLZ945, oligodendrocyte precursor cells are reduced in early, postnatal mouse brains (57). As recently described, tamoxifen, a selective estrogen receptor modulator approved for the treatment of breast cancer and previously linked to a low incidence of retinal toxicity, was unexpectedly found to exert marked protective effects against photoreceptor degeneration. Tamoxifen treatment decreased retinal microglia activation in a genetic (*Pde6b*^rd10^) model of RP and limited the production of inflammatory cytokines and as a consequence reduced microglial-mediated toxicity to photoreceptors (58). Minocycline, a semi-synthetic tetracycline derivative, prevents NF-κB activation by blockade of Toll-like receptor signaling and counteracts microglial release of TNF-α and IL-1β. This is probably why there are also good indications that minocycline is effective in dampening microglial neurotoxicity and to prevent photoreceptor apoptosis (37, 59). Like minocycline, sulforaphane, a naturally occurring isothiocyanate, also inhibits the proteolytic cleavage of NF-κB and inhibits light-induced photoreceptor apoptosis (60). In a similar manner, polysaccharides were effective in preserving photoreceptors against degeneration in rd10 mice partly through inhibition of NF-κB (61).

In conclusion, both strategies, inhibiting microglial activation and/or inhibition of NF-κB-signaling, can provide useful approaches to prevent retinal degeneration in RP.

## Ethics Statement

All procedures concerning animals were performed with permission of the local authority (Regierung von Oberbayern and RP Freiburg).

## Author Contributions

TB, TG, and SM designed research. TB, TG, MK, LA, CS, MBo SP, MP, MBu, MBi, SM, and JW performed experiments, analyzed, and interpreted the data. TG and MK designed the figures. TG, TB, and SM wrote the manuscript. All authors edited the manuscript.

## Conflict of Interest Statement

TG was employed by Synovo GmbH, Tübingen, Germany, and MB was employed by IMGM Laboratories GmbH, Planegg, Germany. All other authors declare no competing interests.
